# The causal effect of switching from precarious to standard employment on mortality in Sweden

**DOI:** 10.1093/eurpub/ckac130.094

**Published:** 2022-10-25

**Authors:** N Matilla-Santander, AA Matthews, V Gunn, C Muntaner, B Kreshpaj, DH Wegman, J Jonsson, T Bodin, PPWR Consortium

**Affiliations:** 1 Unit of Occupational Medicine, Karolinska Institutet, Stockholm, Sweden; 2 Unit of Epidemiology, Karolinska Institutet, Stockholm, Sweden; 3 Lawrence S. Bloomberg Faculty of Nursing, University of Toronto, Toronto, Canada; 4 MAP Centre for Urban Health Solutions, Li Ka Shing Knowledge Institute, Toronto, Canada; 5 Division of Social and Behavioral Sciences, University of Toronto, Toronto, Canada; 6 University of Massachusetts Lowell, Lowell, USA; 7 Centre for Occupational and Environmental Medicine, Stockholm Region, Stockholm, Sweden

## Abstract

**Background:**

Precarious employment (PE) is a well-known social determinant of health and health inequalities, yet the effect of PE on mortality has not been explored sufficiently and high-quality longitudinal studies are lacking. When studying this effect, several methodological factors must be considered, one of them being the immortal time bias or prevalent user bias. A framework that helps us overcome these biases is the target trial. Therefore, the aim of this study is to estimate the causal effect of switching from precarious to standard employment (SE) on the 12-year risk of all-cause mortality among precariously employed workers aged 20-55 in Sweden.

**Methods:**

We emulated the target trial as a series of 11 target trials (starting at any year between 2005 and 2016), such that each individual may participate in multiple trials using Swedish register data (N = 251274). We classified individuals as: a) workers that at baseline (start) move from PE to SE and then followed while in SE or b) continuation of PE over follow-up. All-cause mortality was measured from 2006 to 2017. We pooled data for all 11 emulated trials and used pooled logistic regression to estimate intention-to-treat effects via hazard ratios and standardized survival curves.

**Results:**

The following results are preliminary. Individuals that continued on PE were 185,480 and those that initiated SE were 65,794. Over the 12-year follow-up, 1553 individuals died. The estimated observational analogue of the intention-to-treat 12-year survival difference for all cause-mortality between workers that continued on PE and those that initiated SE was of -0.2%, and the HR:0.82, 95%CI:0.72-0.94.

**Conclusions:**

The following conclusions are preliminary. According to our results, we find indication that shifting from PE to SE decreased the risk of death. Our study highlights the crucial role of decent employment conditions for health.

**Key messages:**

- Changing from precarious to more decent employment conditions decreases the risk of death by any cause in a cohort of Swedish workers.
- This study provides evidence that precarious employment has also an effect on any cause mortality.

